# Supplementary material for: Amphetamine disrupts haemodynamic correlates of prediction errors in nucleus accumbens and orbitofrontal cortex
Source: Neuropsychopharmacology. 2019 Nov 8;45(5):793–803. doi: 10.1038/s41386-019-0564-8 (PMC7075902; doi:10.1038/s41386-019-0564-8)
Supplement: Supplementary file 2 — Supplementary Figures [file 41386_2019_564_MOESM2_ESM.pdf]

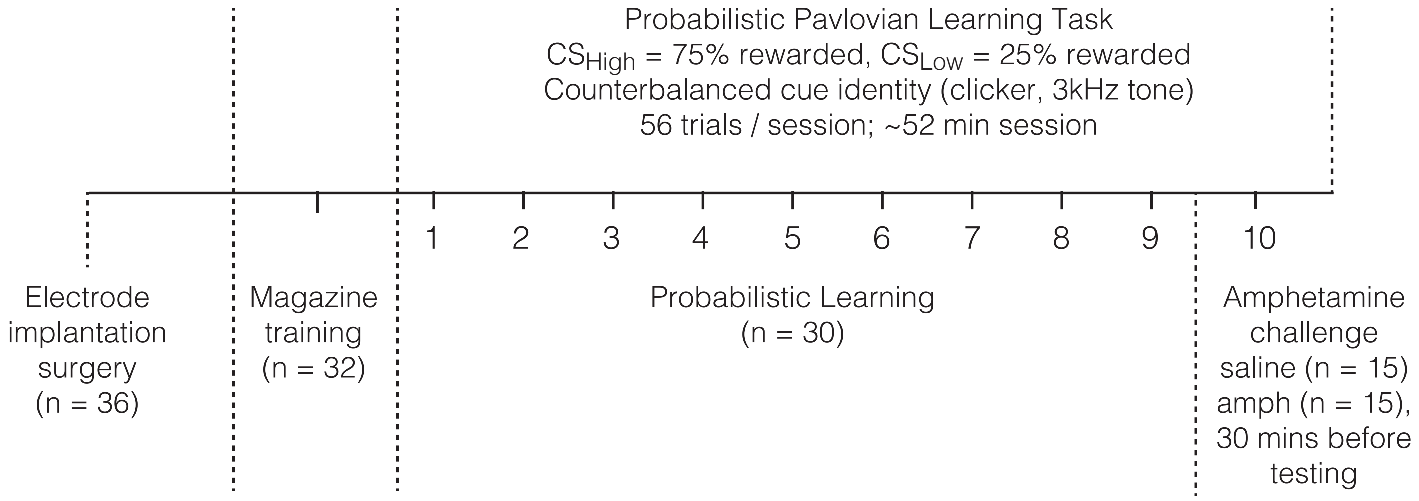
Probabilistic Pavlovian Learning Task
CS_{High} = 75% rewarded, CS_{Low} = 25% rewarded
Counterbalanced cue identity (clicker, 3kHz tone)
56 trials / session; ~52 min session
1
2
3
4
5
6
7
8
9
10
Electrode implantation surgery (n = 36)
Magazine training (n = 32)
Probabilistic Learning (n = 30)
Amphetamine challenge saline (n = 15) amph (n = 15), 30 mins before testing

Figure S1

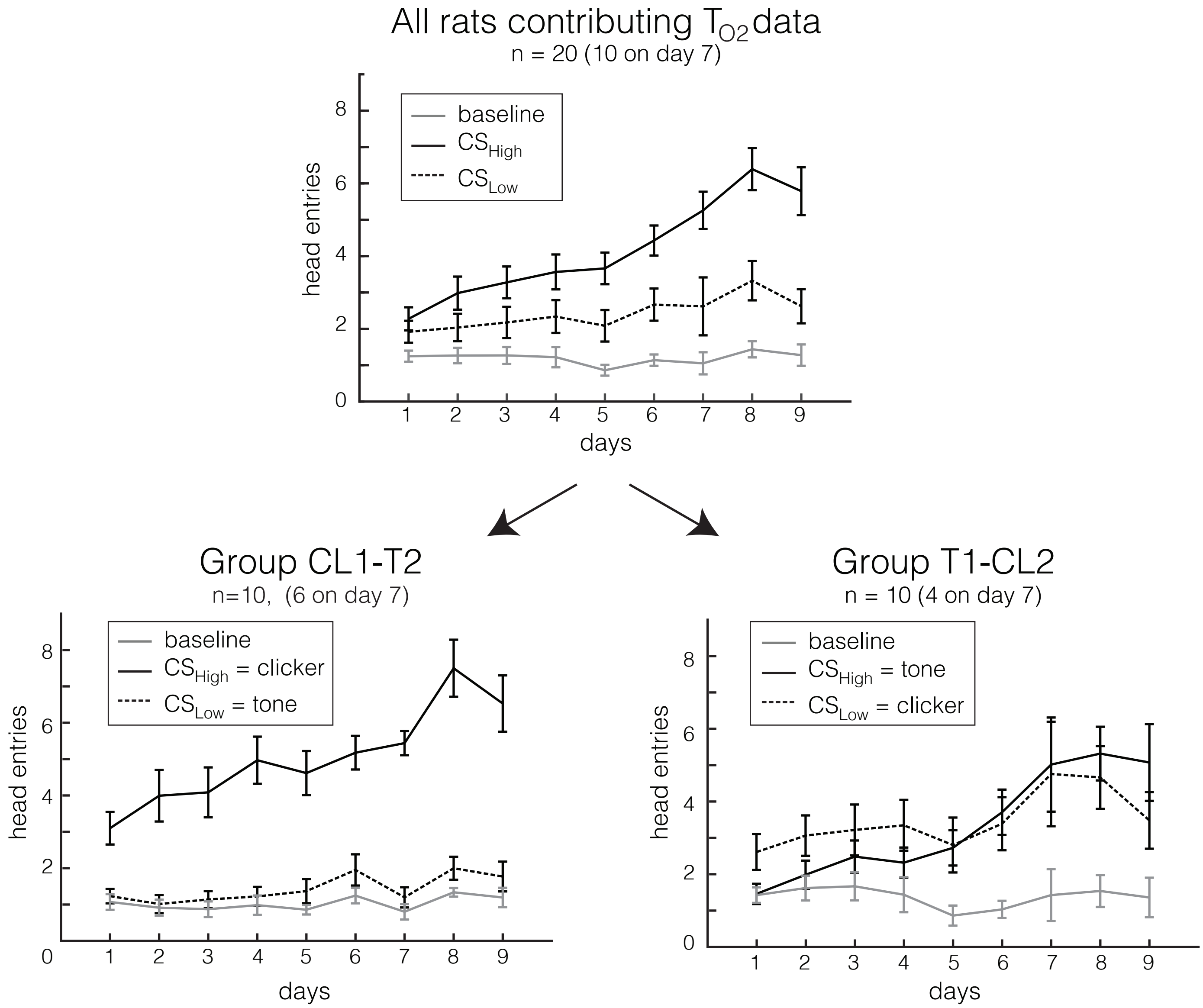

All rats contributing T_{O2} data
n = 20 (10 on day 7)
baseline
CS_{High}
CS_{Low}
head entries
days
Group CL1-T2
n=10, (6 on day 7)
baseline
CS_{High} = clicker
CS_{Low} = tone
head entries
days
Group T1-CL2
n = 10 (4 on day 7)
baseline
CS_{High} = tone
CS_{Low} = clicker
head entries
days

Figure S2

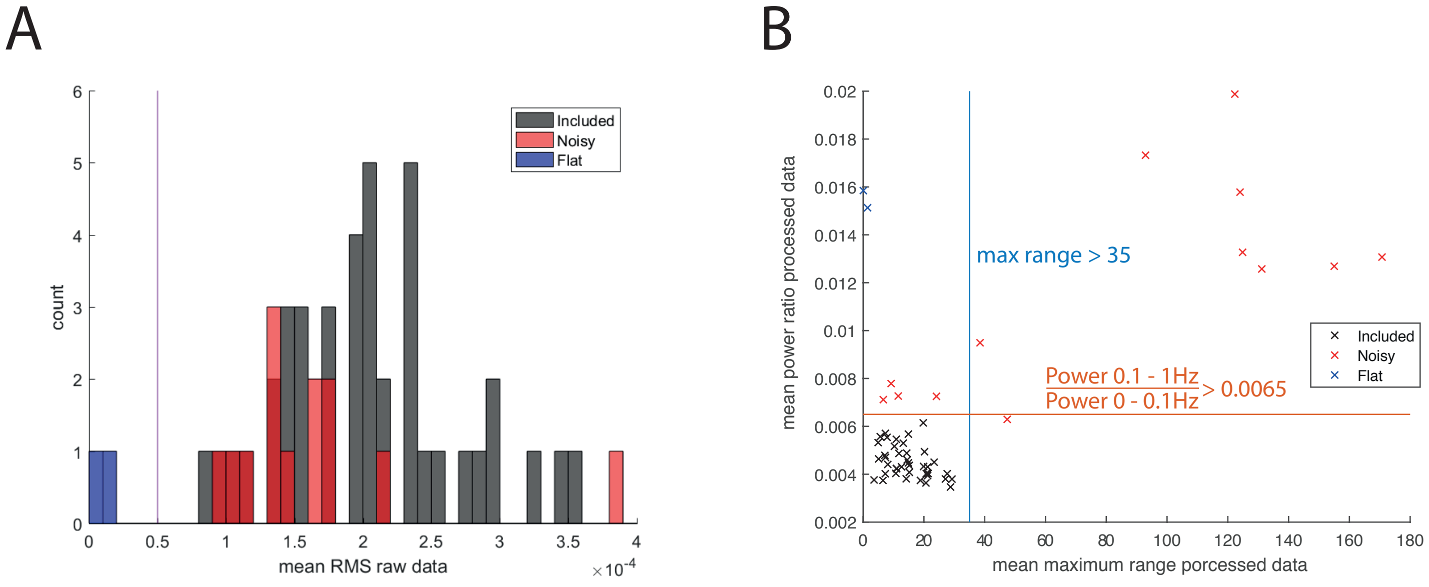
A
B
count
mean RMS raw data
×10^-4
Included
Noisy
Flat
mean power ratio processed data
mean maximum range porcessed data
max range > 35
Power 0.1 - 1Hz
Power 0 - 0.1Hz
> 0.0065

Figure S3

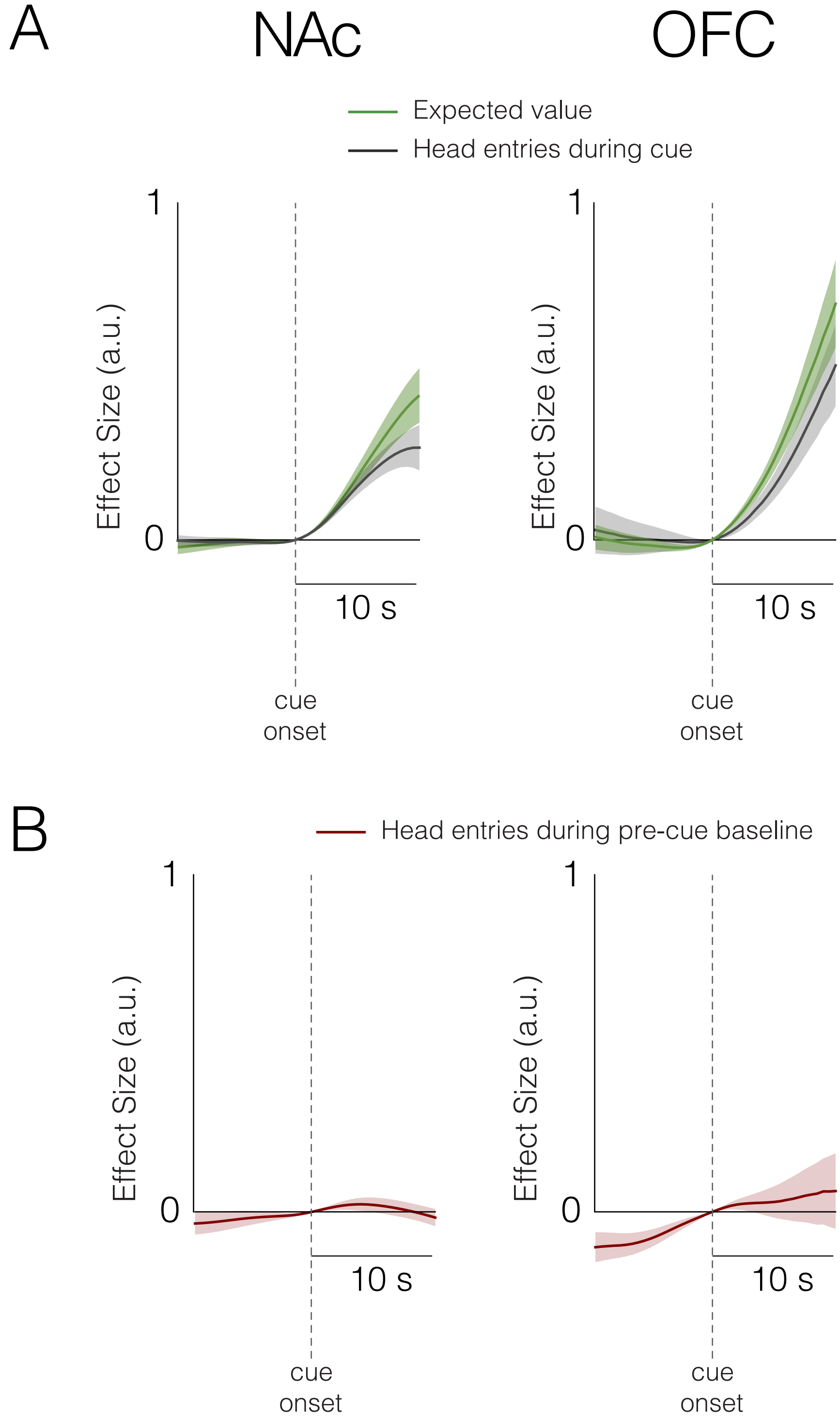
A
NAc
OFC
Expected value
Head entries during cue
1
0
Effect Size (a.u.)
10 s
cue
onset
B
Head entries during pre-cue baseline
1
0
Effect Size (a.u.)
10 s
cue
onset

Figure S4

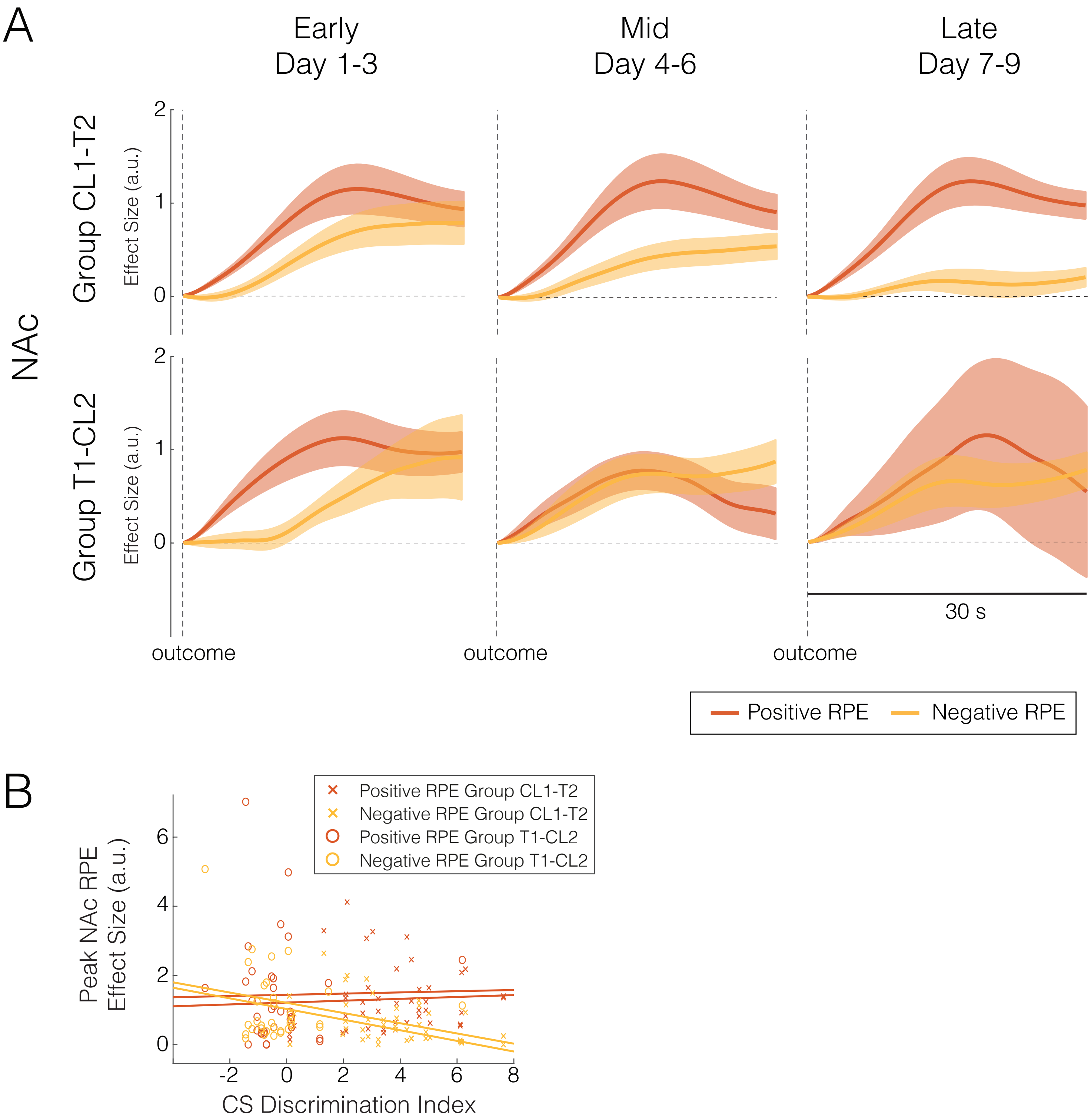
A
Early
Day 1-3
Mid
Day 4-6
Late
Day 7-9
NAc
Group CL1-T2
Group T1-CL2
Effect Size (a.u.)
30 s
outcome
Positive RPE
Negative RPE
B
Positive RPE Group CL1-T2
Negative RPE Group CL1-T2
Positive RPE Group T1-CL2
Negative RPE Group T1-CL2
Peak NAc RPE
Effect Size (a.u.)
CS Discrimination Index

Figure S5

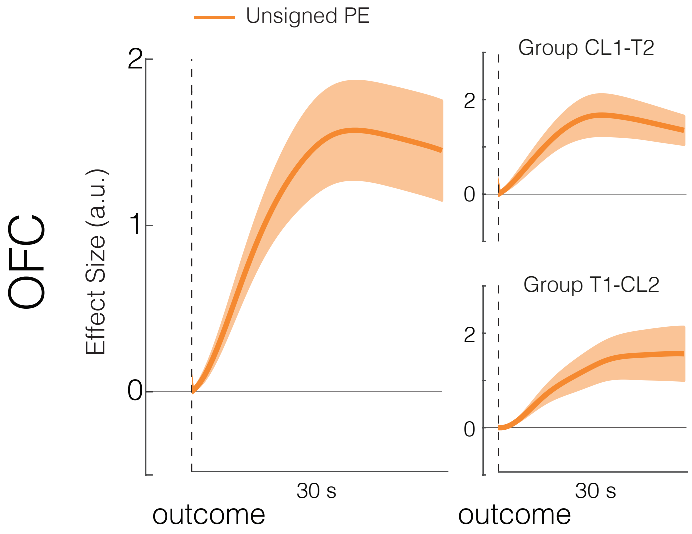
Unsigned PE
OFC
Effect Size (a.u.)
2
1
0
30 s
outcome
Group CL1-T2
2
0
Group T1-CL2
2
0
30 s
outcome

Figure S6

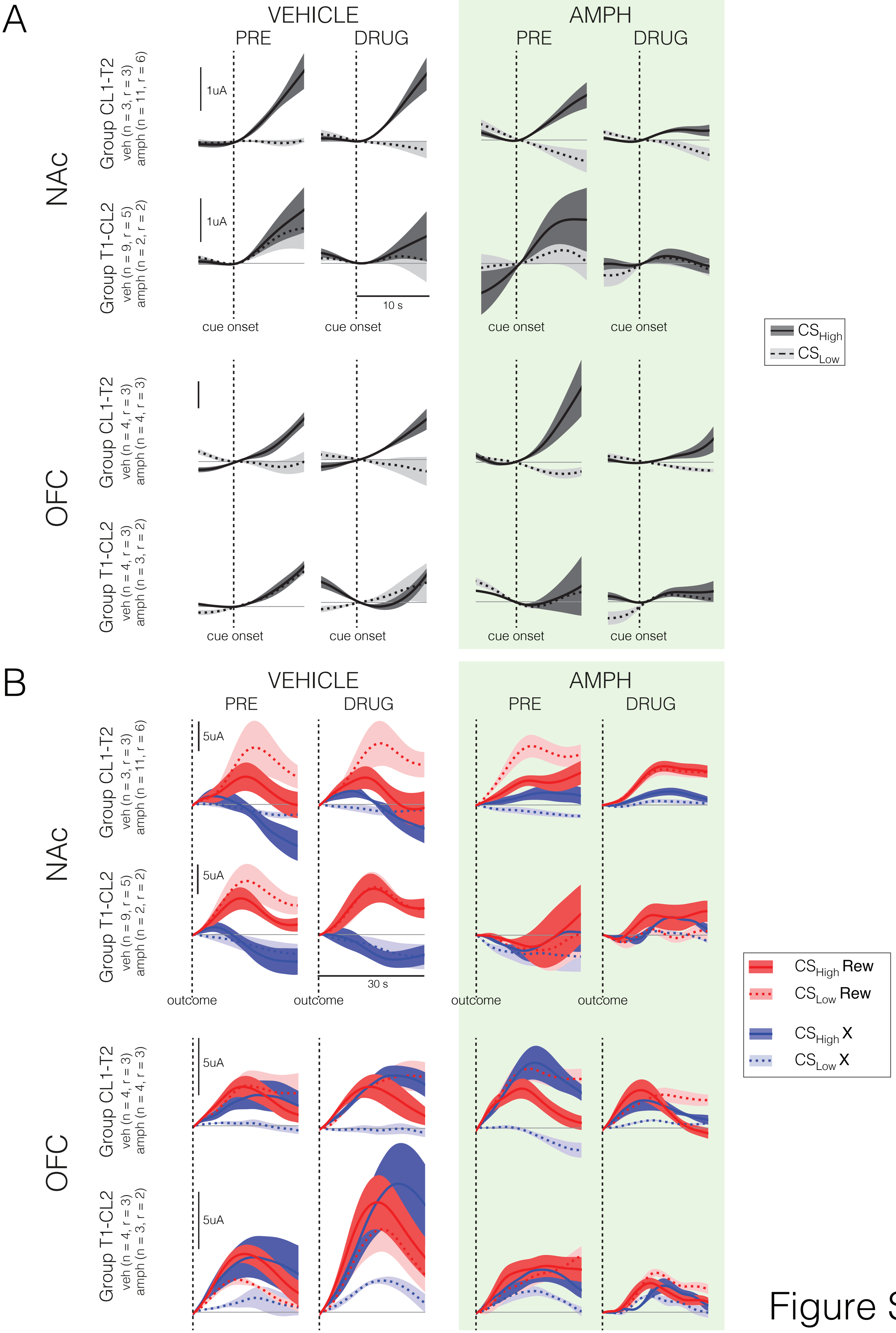
A
VEHICLE
AMPH
PRE
DRUG
NAc
OFC
Group CL1-T2
veh (n = 3, r = 3)
amph (n = 11, r = 6)
Group T1-CL2
veh (n = 9, r = 5)
amph (n = 2, r = 2)
veh (n = 4, r = 3)
amph (n = 4, r = 3)
veh (n = 4, r = 3)
amph (n = 3, r = 2)
1uA
10 s
cue onset
CS$_{High}$
CS$_{Low}$
B
5uA
30 s
outcome
CS$_{High}$ Rew
CS$_{Low}$ Rew
CS$_{High}$ X
CS$_{Low}$ X

Figure S7
